# Contact Tracing for Mpox Clade II Cases Associated with Air Travel — United States, July 2021–August 2022

**DOI:** 10.15585/mmwr.mm7335a1

**Published:** 2024-09-05

**Authors:** Kristin C. Delea, Tai-Ho Chen, Kayla Lavilla, Yonette Hercules, Shannon Gearhart, Leigh Ellyn Preston, Christine M. Hughes, Faisal S. Minhaj, Michelle A. Waltenburg, Brittany Sunshine, Agam K. Rao, Andrea M. McCollum, Kara Adams, Miguel Ocaña, Olubunmi Akinkugbe, Clive Brown, Francisco Alvarado-Ramy

**Affiliations:** ^1^Division of Global Migration Health, National Center for Emerging and Zoonotic Infectious Diseases, CDC; ^2^Doctors Without Borders, Addis Ababa, Ethiopia;^ 3^Division of High-Consequence Pathogens and Pathology, National Center for Emerging and Zoonotic Infectious Diseases, CDC.

**Summary**

**What is already known about this topic?**

Monkeypox virus (MPXV) can spread among persons and cause severe mpox. Before 2021, limited information to assess the risk for MPXV transmission aboard commercial aircraft was available. Two earlier investigations identified no secondary cases among passengers seated near infected travelers.

**What is added by this report?**

During 2021–2022, 113 persons traveled on commercial flights while they were infectious with clade II mpox. Among 1,046 traveler contacts followed by U.S. public health agencies, CDC identified no secondary cases.

**What are the implications for public health practice?**

Traveling on a flight with a person with mpox does not appear to constitute an exposure risk or warrant routine contact tracing activities.

## Abstract

Monkeypox virus (MPXV) can spread among humans through direct contact with lesions, scabs, or saliva; via respiratory secretions; and indirectly from fomites; via percutaneous injuries; and by crossing the placenta to the fetus during pregnancy. Since 2022, most patients with mpox in the United States have experienced painful skin lesions, and some have had severe illness. During 2021–2022, CDC initiated aircraft contact investigations after receiving reports of travelers on commercial flights with probable or confirmed mpox during their infectious period. Data were collected 1) during 2021, when two isolated clade II mpox cases not linked to an outbreak were imported into the United States by international travelers and 2) for flights arriving in or traveling within the United States during April 30–August 2, 2022, after a global clade II mpox outbreak was detected in May 2022. A total of 113 persons (100 passengers and 13 crew members) traveled on 221 flights while they were infectious with mpox. CDC developed definitions for aircraft contacts based on proximity to mpox cases and flight duration, sent information about these contacts to U.S. health departments, and received outcome information for 1,046 (68%) of 1,538 contacts. No traveler was found to have acquired mpox via a U.S. flight exposure. For persons with mpox and their contacts who had departed from the United States, CDC forwarded contact information as well as details about the exposure event to destination countries to facilitate their own public health investigations. Findings from these aircraft contact investigations suggest that traveling on a flight with a person with mpox does not appear to constitute an exposure risk or warrant routine contact tracing activities. Nonetheless, CDC recommends that persons with mpox isolate and delay travel until they are no longer infectious. 

## Introduction

Monkeypox virus (MPXV) can spread among humans through direct contact with lesions, scabs, or saliva; via respiratory secretions; indirectly from fomites; via percutaneous injuries; and by crossing the placenta to the fetus during pregnancy (*1*). Mpox is a disease caused by infection with MPXV. Since May 2022, approximately 33,000 mpox cases have occurred in the United States*; most patients have experienced painful skin lesions, typically in the anogenital region, and some have suffered life-threatening complications or protracted illness. Before 2021, mpox cases outside of Africa occurred in a U.S. zoonotic outbreak involving imported wild African rodents in 2003 and in a limited number of travelers infected before travel from West Africa. No cases of transmission attributed to aircraft cabin exposure from these travelers have been reported (*2*). In 2021, two travelers flew on commercial flights into the United States while infectious with mpox (*3*,*4*). Then, in May 2022, a large global outbreak of MPXV clade IIb was recognized, primarily associated with male-to-male sexual contact. This outbreak has affected more than 110 countries, and hundreds of persons have traveled via commercial aircraft while infectious with mpox (*5*). Because of concerns for the potential for in-flight transmission, CDC initiated aircraft contact investigations after receiving reports of persons with probable or confirmed mpox traveling on commercial flights during the infectious period. This report describes findings from these investigations.

## Methods

### Data Source

This report includes data on aircraft contact investigations initiated by CDC for persons with mpox^†^ who were identified by U.S. public health departments as having traveled on domestic or arriving international flights while infectious. These mpox cases occurred in 2021 or during April 30–August 2, 2022. The infectious period was defined as the period commencing with the onset of illness through such time that all lesions had crusted over, the crusts had separated, and a fresh layer of healthy skin had formed under the crust.

### Notification of Potential Exposure

CDC is notified when a U.S. or a foreign public health agency learns that persons with certain communicable diseases of public health concern were infectious while traveling on a commercial aircraft. Based on disease-specific protocols (CDC, unpublished data, 2019), CDC determines whether the criteria to initiate an aircraft contact investigation have been met. If the criteria are met, CDC sends a traveler manifest request to the airline, enabling identification of potentially exposed passengers and crew members.

CDC adapted the mpox community exposure risk assessment^§^ to define an exposure risk zone for aircraft contact investigations. In general, air passengers seated within a 3-foot radius (one seat in any direction) of the potentially infectious person on flights of ≤3 hours’ duration or within a 6-foot radius (two seats in any direction) on flights of >3 hours’ duration were considered to be in the exposure risk zone. For two flights in July 2021 involving an infectious passenger who had an extensive purulent rash, the exposure risk zone was expanded to include all passengers who had potentially used the same lavatory (*3*).

Crew members serving the cabin where infectious passengers had been seated were considered contacts of those patients. If crew members were determined to be infectious while on duty, fellow crew members who worked the same flights for >3 cumulative hours were also classified as having had an exposure. Passengers were not considered contacts of infected cabin crew members because crew members typically wore gloves (standard procedure while distributing or retrieving items) and masks (customary practice during the COVID-19 pandemic); also, direct interactions with any individual passenger were likely brief.

### Notification per International Health Regulations

CDC used information from the flight manifests provided by airlines to identify travelers seated in the exposure risk zone and obtain their contact information, the latter supplemented by federal and third-party databases. CDC shared this information securely with U.S. or foreign public health agencies in jurisdictions of the travelers’ residence to enable contact tracing and symptom monitoring for 21 days after flight (the maximum known incubation period for mpox).

In accordance with the 2005 International Health Regulations (IHR), CDC sent notifications to other countries, via their National Focal Points, about any potentially infectious persons aboard flights departing the United States and any aircraft contacts who traveled to their countries (*6*). This activity was reviewed by CDC, deemed not research, and was conducted consistent with applicable federal law and CDC policy.^¶^

## Results

### Contacts of Airline Passengers with Mpox Not Linked to an Outbreak (2021)

During 2021, two isolated confirmed clade II mpox cases not linked to an outbreak were imported into the United States by international travelers who had traveled on three commercial flights (3,4). CDC received individual outcome information for all 149 aircraft contacts (138 passengers and 11 crew members) from the 30 domestic health departments that had conducted public health follow-up (Table). No secondary mpox cases were reported.

**TABLE:** Aircraft contacts of persons with probable or confirmed mpox on commercial flights into or within the United States and outcomes as reported by U.S. jurisdictions, July 2021–August 2022

Data elements and risk assignments considered in contact tracing	Reporting period, no. (%)			
	Jul 2021*	Nov 2021	Apr–Aug 2022	Total
Mpox cases	1	1	111	**113**
Flights	2	1	218	**221**
Aircraft passenger contacts^†^	129	9	796	**934**
Aircraft crew contacts^§^	11	0	593	**604**
Travelers for whom FPHNs were sent^¶^	56	22	221	**299**
U.S. jurisdictions assigned contacts	26	4	51	**51**
Contacts with outcomes reported to CDC	140 (100)	9 (100)	897 (65)	**1,046 (68)**

**Abbreviation**: FPHN = foreign public health notification.

* For two flights in July 2021 involving an infectious passenger who had an extensive purulent rash, the exposure risk zone was expanded to include all passengers who had potentially used the same lavatory.

^†^ Passengers were considered aircraft contacts if seated within two seats of an infected traveler for flights >3 hours, or within one seat for flights ≤3 hours, in any direction.

^§^ Crew either serving infected travelers or working with an infected crew member for >3 hours were classified as having an exposure.

^¶^ An FPHN is a notice sent by CDC to another country through its National International Health Regulations Focal Point pursuant to Article 44 of the 2005 International Health Regulations, which calls for collaboration among countries in the detection, assessment, and response to events of potential public health significance. https://pubmed.ncbi.nlm.nih.gov/27166578

### Contacts of Airline Passengers with Outbreak-Related Mpox (2022)

In 2022, a total of 111 persons with probable or confirmed clade II mpox linked to the global outbreak who traveled on commercial aircraft while infectious were identified. Among 1,389 identified aircraft contacts, CDC received aggregate outcome information from 30 U.S. health departments about 897 (65%) (884 passengers and 13 crew members), who traveled on 218 commercial flights during April 30–August 2. None of the aircraft contacts was reported to have developed mpox during symptom monitoring.

### CDC Notification of National Focal Points

CDC notified National IHR Focal Points in 41 countries about 299 international travelers. This group included 84 persons with mpox and 215 exposed contacts of persons with infectious mpox on flights, all of whom traveled to these countries before or after their U.S. arrival. Among the 21 National IHR Focal Points that provided outcome information on contact tracing to CDC, one reported that an aircraft contact had developed symptoms within the 21-day monitoring and received a diagnosis of mpox. CDC does not have further details, including seating proximity to the infected traveler or case investigation data (including any potential community exposures) to assess risk factors beyond aircraft exposure. The original report came from a subnational agency, and the reporting country’s privacy laws did not permit further inquiry.

## Discussion

U.S. health departments reported no cases of mpox attributed to flight exposures during the U.S. public health follow-up of 1,046 passengers and crew members identified as aircraft contacts during July 2021–August 2022. One case of mpox in a traced aircraft contact was reported in a non-U.S. resident by a public health authority in another country; however, the epidemiologic information provided was insufficient to ascertain the likelihood that transmission occurred during the flight. 

These findings are consistent with those from an investigation of mpox exposures among aircraft contacts conducted by Australia’s Victorian Department of Health involving 15 international flights occurring during May–October 2022 (*7*). Australian investigators used a broader definition to identify exposed passengers, whereby travelers seated beyond two seats in any direction were included as contacts. Australian public health officers did not identify any secondary cases of mpox among the flight contacts, either monitored or unmonitored (i.e., using other means to link cases to the flights). Available evidence suggests that the risk for acquiring mpox during air travel, even among travelers exposed to persons with infectious cases, is very low. This very low risk could be attributed, at least in part, to the unlikely occurrence on flights of the direct contact with mpox lesions that is associated with most secondary cases (*1*). Based on available information, including preliminary analyses of these data, CDC discontinued routine aircraft contact investigations for mpox in August 2022.

Relative to clade II mpox, clade I infections have historically been associated with increased transmissibility (*8**,**9*). However, both clade I and clade II mpox spread in the same ways, primarily via close physical or intimate contact with infected lesions and less often via infectious respiratory secretions and fomites. The type of contact most often associated with secondary cases (e.g., sex or sharing bedding) is unlikely to occur on aircraft. Limited aircraft contact investigations could be considered for the first probable or confirmed clade I MPXV infections identified in recent air travelers to corroborate equivalent risk with clade II.

### Limitations

The findings in this report are subject to at least three limitations. First, outcome data were missing for approximately one third of identified aircraft contacts. This shortcoming might be accounted for by factors such as competing priorities for resources at state, local, and territorial health departments; inaccurate or incomplete traveler contact information; and nonresponsive travelers. Second, passengers were not considered contacts if the patient was a crew member, which might have led to identification of fewer contacts. Finally, although the findings presented in this report might apply to MPXV irrespective of clade, the cases all involved clade II MPXV. 

### Implications for Public Health Practice

Aircraft contact investigations are complex and resource-intensive endeavors that can divert resources from other public health activities with higher prevention yield, particularly during a large outbreak response. This report provides the largest published series of mpox aircraft contacts and suggests a very low risk for clade II MPXV transmission in the commercial aircraft cabin setting. Traveling on a flight with a person with mpox caused by clade II MPXV does not appear to constitute an exposure risk or warrant routine contact tracing activities. The criteria for conducting aircraft contact investigations should be subject to continuous evaluation based on the most current scientific evidence to guide public health risk assessments and interventions. CDC continues to recommend that persons with mpox isolate and delay travel until they are no longer infectious.**

## Footnotes

*
https://www.cdc.gov/poxvirus/mpox/response/2022/mpx-trends.html

† Persons with mpox defined per U.S. case definitions: https://www.cdc.gov/poxvirus/mpox/clinicians/case-definition.html.

§
https://www.cdc.gov/poxvirus/mpox/clinicians/monitoring.html

¶ 45 C.F.R. part 46.102(l)(2), 21 C.F.R. part 56; 42 U.S.C. Sect. 241(d); 5 U.S.C. Sect. 552a; 44 U.S.C. Sect. 3501 et seq.

**
https://www.cdc.gov/poxvirus/mpox/if-sick/what-to-do.html
